# Safety and Efficacy of Cariprazine in a Subset of Patients with Bipolar I Disorder: A Phase IV Study

**DOI:** 10.1192/j.eurpsy.2026.11199

**Published:** 2026-07-31

**Authors:** C. S. Jilowa, N. Vemana, M. Bhirud, A. Dharmadhikari, D. Chaudhari, Y. Patel, P. Dasud, R. Khandelwal, S. K. Padhy, S. Gopal, L. Lakhwani, S. saha, D. Patil, S. Behera, S. Pandit, P. Ghadge, S. Mehta

**Affiliations:** 1 Jawahar Lal Nehru Medical College, Ajmer; 2 Excel Hospital, Secunderabad; 3 Dhadiwal Hospital Incoalition with Shreeji Healthcare, Nashik; 4 Shree Aashirwaad Hospital, Dombivili; 5 GSVM Medical College, Kanpur; 6 VS General Hospital, Ahmedabad; 7 Calida Psychiatric Hospital, Raigad; 8 Apex hospital Pvt Ltd, jaipur; 9 All India Institute of Medical Sciences, Bhubaneswar; 10 S.P. Medical College & A.G. of Hospitals, Bikaner; 11 Sun Pharma laboratories limited, Mumbai, India

## Abstract

**Introduction:**

Cariprazine is an atypical antipsychotic drug, demonstrating efficacy across manic, depressive, and mixed episodes in bipolar disorder with a favourable safety profile making it a good therapeutic option in patient management. A Phase IV, prospective, multi centre, single arm study was conducted to evaluate safety and efficacy of cariprazine in Indian patients with Schizophrenia and Acute Treatment of Manic or Mixed Episodes Associated with Bipolar I Disorder.

**Objectives:**

The Safety and Efficacy of Cariprazine capsules was evaluated in the subset of patients with Acute Treatment of Manic or Mixed Episodes Associated with Bipolar I Disorder in the Phase IV, single arm study.

**Methods:**

Patients with Bipolar I disorder with, manic or mixed type, with or without psychotic symptoms, based on the DSM-V were enrolled in the study with Young Mania Rating Scale (YMRS) Total score ≥20 and Montgomery–Asberg Depression Rating Scale (MADRS) Total score <18. A total 75 patients, in the age range of 18 to 65 years, received Cariprazine starting dose 1.5 mg with further up titration to 3 to 6 mg for 3-weeks. Primary Endpoint was safety evaluation and secondary Endpoints were change in YMRS score, Clinical global impression -Bipolar version (CGI-BP) score and MADRS score. **[Clinical Trial Registration: [CTRI/2023/05/052793]**

**Results:**

A total of 15 TEAEs (Treatment emergent Adverse Events) were reported in 14 patients and insomnia (3 events) followed by, diarrhoea, vomiting, headache, and cough (2 events each) were most common. All events were grade 1 (mild) and resolved.

With cariprazine treatment continued, there is gradual improvement in study patients based on changes in YMRS CGI-BP, and MADRS scores. Mean (SD) change in YMRS scores were -9.97 (±6.76) on Day 21 (p<0.0001) demonstrating efficacy in alleviating manic symptoms. Mean (SD) changes in CGI-BP scores were -4.80 (±4.54) on Day 21 (p-values: <0.0001) leading to overall reduction in illness severity and improvement in global functioning. Mean (SD) changes in MADRS scores were -2.83 (±3.16) on Day 21 showing improvement in depressive symptoms and overall mood stabilization (p<0.0001).

**Conclusions:**

Cariprazine capsules have demonstrated in managing clinical symptoms of Bipolar I disorder, with balanced efficacy in managing mania and bipolar depression in bipolar 1 disorder. The study treatment was found to be safe and well tolerated.

**Disclosure of Interest:**

None Declared

